# Integrated agriculture programs to address malnutrition in northern Malawi

**DOI:** 10.1186/s12889-016-3840-0

**Published:** 2016-11-28

**Authors:** Rachel Bezner Kerr, Emmanuel Chilanga, Hanson Nyantakyi-Frimpong, Isaac Luginaah, Esther Lupafya

**Affiliations:** 1 Department of Development Sociology, Cornell University, New York, USA; 2 University of Livingstonia, Livingstonia, Malawi; 3 The Integrative Agroecology Group, University of Toronto, Scarborough, Canada; 4 Department of Geography, Western University, London, Canada; 5 SFHC Organization, Ekwendeni Hospital, Ekwendeni, Malawi

**Keywords:** Undernutrition, Gender, Masculinities, Participatory Research, Malawi

## Abstract

**Background:**

In countries where the majority of undernourished people are smallholder farmers, there has been interest in agricultural interventions to improve nutritional outcomes. Addressing gender inequality, however, is a key mechanism by which agriculture can improve nutrition, since women often play a crucial role in farming, food processing and child care, but have limited decision-making and control over agricultural resources. This study examines the approaches by which gender equity in agrarian, resource-poor settings can be improved using a case study in Malawi.

**Methods:**

A quasi-experimental design with qualitative methods was used to examine the effects of a participatory intervention on gender relations. Thirty married couple households in 19 villages with children under the age of 5 years were interviewed before and then after the intervention. An additional 7 interviews were conducted with key informants, and participant observation was carried out before, during the intervention and afterwards in the communities. The interviews were recorded and transcribed, and analysed qualitatively for key themes, concepts and contradictions.

**Results:**

Several barriers were identified that undermine the quality of child care practices, many linked to gender constructions and norms. The dominant concepts of masculinity created shame and embarrassment if men deviated from these norms, by cooking or caring for their children. The study provided evidence that participatory education supported new masculinities through public performances that encouraged men to take on these new roles. Invoking men’s family responsibilities, encouraging new social norms alongside providing new information about different healthy recipes were all pathways by which men developed new ‘emergent’ masculinities in which they were more involved in cooking and child care. The transformational approach, intergenerational and intra-gendered events, a focus on agriculture and food security, alongside involving male leaders were some of the reasons that respondents named for changed gender norms.

**Conclusions:**

Participatory education that explicitly addresses hegemonic masculinities related to child nutrition, such as women’s roles in child care, can begin to change dominant gender norms. Involving male leaders, participatory methods and integrating agriculture and food security concerns with nutrition appear to be key components in the context of agrarian communities.

## Background

Recent development scholarship has focused on integrating agricultural interventions and nutrition education to improve nutrition in agrarian settings. Several reviews have concluded that, thus far, agricultural interventions have shown weak, limited evidence of improving nutritional outcomes in the Global South [1–3]. Although inadequate dietary intake and morbidity are immediate causes of undernutrition, studies have shown that household gender inequality relates to high rates of child undernutrition [4, 5]. Women’s empowerment, in terms of control over resources, decision-making and more equitable workloads are all pathways by which nutrition can be improved through agricultural interventions [6, 7]. What are the processes and approaches that can improve gender equity in agrarian, resource-poor settings? The purpose of this paper is to explore this question using a case study of a participatory agriculture and nutrition education program in northern Malawi, a setting with both high levels of under nutrition and gender inequality. A quasi-experimental research design was used to address three research questions: 1) What is the current household division of labour and decision-making? 2) What are the local explanations for this division of labour and decision-making? 3) Can recipe days influence this household division of labour and decision-making? The case is made for participatory nutrition education that supports emergent new masculinities to foster change in gender relations and child care. The term ‘care’ as used in child nutrition refers to the behaviours and practices of caregivers who provide the food, health care, stimulation and emotional support necessary for child’s healthy growth and development [8].

Child malnutrition is a major public health challenge in Sub-Saharan Africa, and increases the risk of death, infection and poor cognitive development [9]. Current estimates are that 41% of Malawian children are stunted, 17% are underweight and 5% are wasted [10]. A number of studies in Malawi have also shown that young children’s complementary foods are inadequate in energy density, protein and micronutrients required for child nutrition [11]. As well, low dietary diversity, food insecurity, inadequate number of feedings in a day, low levels of exclusive breastfeeding and seasonal fluctuations in food prices are some of the persistent reasons for higher rates of child undernutrition in Malawi [12, 13]. The majority of Malawians are rural, smallholder farmers who rely on their own food production for their consumption, and experience ongoing challenges with low and declining soil fertility. Malawi has high rates of rural poverty, and chronic seasonal food insecurity is commonly experienced [10, 12].

Gender is a social category which includes roles, responsibilities and ideas about what characteristics make a man and a woman, and gender relations the world over are dynamic, complex and fluid. Gender inequality, or differences experienced between men and women based on gender, is context-specific and multidimensional, and can include unequal rights to employment, property, education, violence perpetrated due to ones’ gender, and unequal workloads based on gender [14]. Gender inequality is a major challenge facing rural households in Malawi, with women having lower educational and employment opportunities, higher levels of workload, experience high levels of domestic violence and lower levels of decision-making and control over assets [10, 15]. Such inequality has implications for maternal nutrition as well as their ability to provide adequate child care to young children [5]. The authors of this paper take an explicitly feminist postcolonial stance [16] in that unequal power relations are attempted to be revealed, understood and challenged, in order to change these relations [17]. At the same time, the authors’ acknowledge that there can be cultural and racially-driven biases about gender difference, that there are a broad range of cultural ideas about gender, and that there are possibilities for multiple layers of inequalities – race, class, gender, position- to shape ideas about ‘improved’ gender relations - which must be taken into account [17–19]. In this paper we use theoretical approaches drawn from feminist theory and critical men’s studies on health [20, 21] to examine a program which integrated agriculture, nutrition and gender. In doing so, we try to open up the ‘black box’ of processes that can lead to improved gender relations – meaning one in which women and men have a more equal say, control over resources, workload and are free from violence and exploitation.

Some nutrition scholars advocate for a transformational education approach which takes into consideration sociocultural factors that affect child care practices. This educational approach is in contrast with an “information-transmission” approach which does not take into consideration local knowledge but has a standardized set of facts to impart [22, 23]. Studies have shown that complementary feeding nutrition education can significantly improve child care and energy intake which can translate into healthy child nutritional status [24–26].

At present, there are few studies that focus on the impact of participatory nutrition education on intra-household gender roles that affect care practices [24–26]. This study thus contributes to the recent efforts to integrate agricultural interventions and nutrition by examining the effects of participatory nutrition education that explicitly addresses gender relations on gender roles and child care. We argue that participatory methods that integrate gender issues and new emergent masculinities into broader agriculture-nutrition approaches show potential for transformative change. The paper is organized as follows. We first provide an overview of the literature on gender, care and nutrition. Next, we describe the case study area before outlining our research methodology. We subsequently present and discuss our qualitative findings in the context of the available literature. We conclude by acknowledging the limitations of our study, and highlighting what our findings mean for efforts to improve child care and nutrition in sub-Saharan Africa.

### The agriculture-gender-nutrition nexus and emergent masculinities

According to studies conducted by UNICEF, it has been recognized that in addition to health care and food security, care for children is vital for child survival, growth and development [27]. In low-income countries, the peak incidence of growth faltering, micronutrient efficiencies and infectious diseases occurs mostly in children between 6 to 24 months, thereby making this a vulnerable and critical time for good child care [9]. Care practices of young children have been found to contribute significantly to child nutritional outcomes [28], and women’s workloads are a major factor in affecting women’s ability to provide high quality child care. Therefore, to effectively address child malnutrition, there is a need to address issues of gender equity and care practices during the complementary feeding period [29]. Most child nutrition intervention programs focus their educational efforts on mothers [30]. Since mothers already have multiple roles in the households, these intervention programs can negatively affect their workload, without addressing gender inequalities that affect child care, such as the division of labour and decision-making [4].

Focusing on mothers also ignores the role in decision-making that men and older women (e.g. grandmothers) often play in early childcare [22, 31]. There has been limited research on the impact of integrating men or other key decision-makers within the extended family, such as grandmothers, into nutrition education programs [22, 23, 32] or the effects of different educational approaches to nutrition. At the same time, recent scholarship has identified the vulnerabilities that men face in resource-poor settings such as Malawi, and the strain that men face in trying to fulfill their roles as providers in such settings [21].

Current studies indicate that in northern Malawi, women are responsible for the vast majority of child care while having limited control over household food resources, competing and heavy work demands, including agriculture [15]. The triple role of women reduces their time for child care, which is likely to negatively affect the nutritional status of children. There are several competing theories for this unequal division of household labour, including the prevailing gender ideology, differences in resource allocation, educational level or prestige [33]. The relative economic resource model suggests that differences in pay influence the amount of household work that men and women do [34], while other studies suggest that educational level, amount of prestige associated with a job, or differences in gender ideology are more important in determining the division of household labour [35]. None of these theories accounts for gender differences in rural agrarian households, and most research on the gender division of labour have been conducted in North America and Europe. A recent systematic review of studies on agriculture, time use and nutritional outcomes found only 89 published studies that fit this description, of which 19 focused on Africa, and 5 were explicitly focused on an agricultural intervention [36]. More research needs to be done on gender division of labour and decision-making in the African context, particularly how time use relates to agriculture and nutritional outcomes [36].

At the same time, there is need for further research on how to promote transformational change of this inequality. Finding ways of motivating fathers to work hand-in hand with mothers in both productive and reproductive tasks could have a positive impact on the nutritional status and general wellbeing of children. Fathers could prepare food and feed the child while mothers are doing other productive work or fathers could do other domestic work while mothers take care of the child. This paper takes the ‘critical men’s studies’ frame, a feminist sociological approach, in which hegemonic masculinities are conceptualized as historically specific, normative patterns of practice that reinforce the subjugation of women to men [21, 37, 38]. While only a few men may actually promote hegemonic masculinities in a given place and time, complicit masculinities reinforce them [38], while heterogeneous and contradictory dimensions of masculinity, alongside efforts to reduce gender inequalities have led to a ‘crisis in masculinity’ [39].

The history of slave and ivory trade, colonial and post-colonial regimes in Malawi instated stark divides in gender roles and responsibilities, in part due to men’s forced migration to mines and plantations, women’s reduced access to and ownership of land and colonial ideas about gender that was inscribed in law [15, 40–42]). Hegemonic masculinities in this region emphasized men as heads of household, strong providers and heavy alcohol users [43]. Domestic spaces in this cultural milieu were decidedly feminine [20]. Recent scholarship on masculinities in southern and eastern Africa, however, show evidence that men living in poverty-striken regions with high levels of unemployment face severe challenges in maintaining hegemonic masculinities, leading to fractured, hybrid, contradictory and shifting masculinities, with an emphasis on male control, power and violence [20, 43–48]. It is in this context that the Soils, Food and Healthy Communities (SFHC) project has been working to change gender relations using participatory methods.

### The research setting and the agriculture-nutrition intervention

**Fig. 1:**
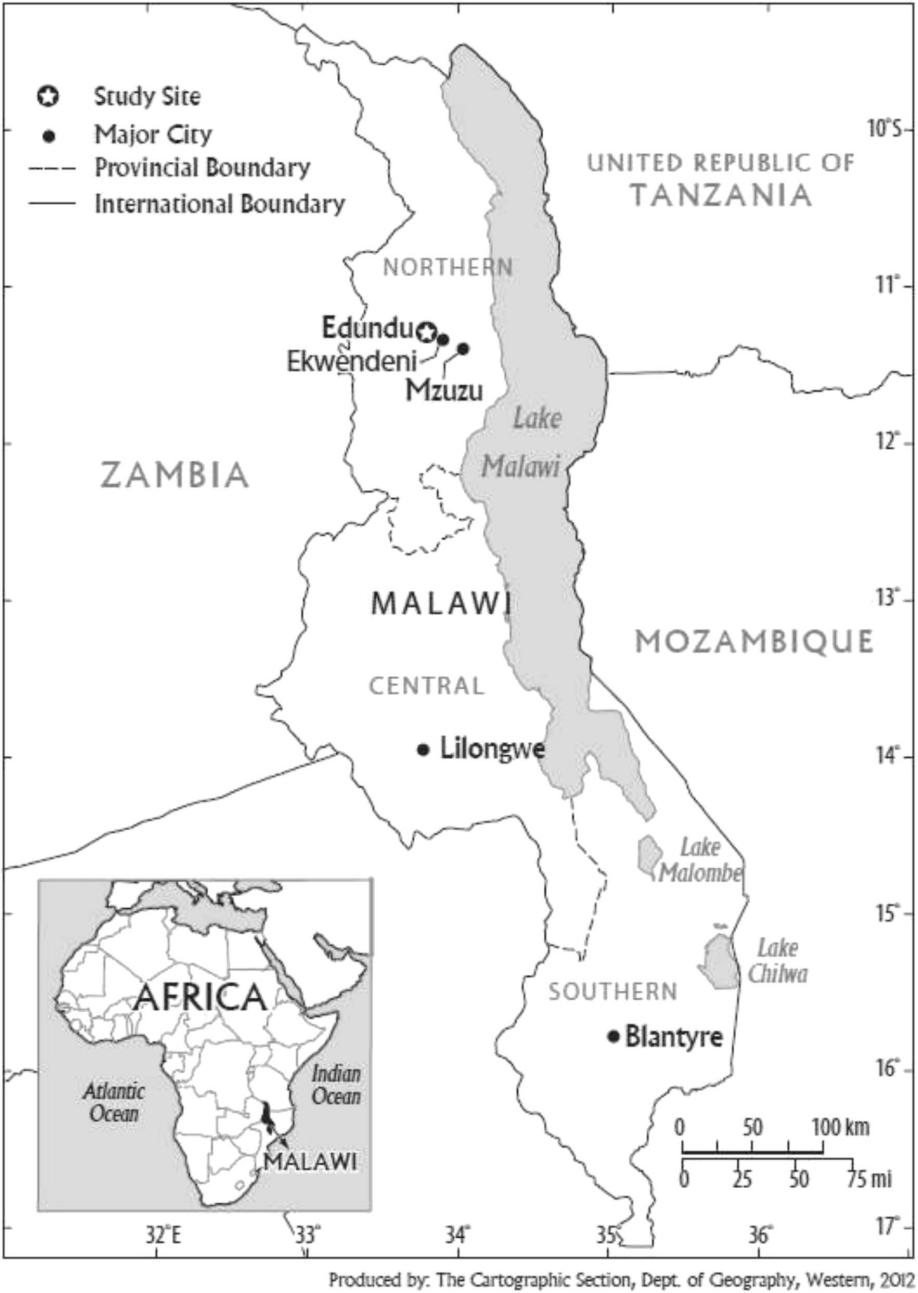
Location of the study area

Malawi is a small landlocked country in southern Africa, bordered by Mozambique, Tanzania and Zambia. It has a current estimated population of 16.7 million, approximately 85% of whom rely on agriculture for incomes and food security [49]. Smallholder agriculture is a major component of the local economy, with key crops including maize, cassava, beans, groundnuts, cowpea, sweet potatoes, several green leafy vegetables, and tobacco as a major cash crop. This research was conducted in the area around Ekwendeni in the northern part of the country. Ekwendeni is a small trading town located approximately 20 km north of Mzuzu city in Malawi (Fig. 1). The town has Ekwendeni Hospital, which offers both curative and primary health care to over 70,000 people residing within its catchment area [31]. About one-third of the smallholder families in the area experience food insecurity every year. Child malnutrition rates are similar to the national average.

The Soils, Food and Healthy Communities (SFHC) project, was initiated by a hospital and researchers in 2000 with a focus on food insecurity and health in Malawi. The SFHC project integrates sustainable agriculture and nutrition using a participatory approach to improve child nutrition, in which farming households develop and test different strategies, including crop diversification, organic methods, and nutrition education [50]. Previous studies found evidence that SFHC participating households improved their food security, nutrition and ecosystem services [51–53].

The SFHC project is based on an ecohealth model which takes into account the interrelationship of ecological, social and economic factors to improve human health [54]. Farmer researchers tested different organic methods to improve their food security and nutrition on their own farm. Participatory workshops, regular monthly meetings of farmer researchers alongside quantatitive and qualitative research methods, were used to assess change over time [55]. The project initially focused on legume intercropping (such as pigeonpea and groundnuts) rotated with maize to improve soil fertility and increase family dietary diversity and later added tubers such as cassava and sweet potatoes. The participatory approach used by SFHC included farmer-led experimentation and research, small discussion groups, field days, farmer exchanges and other methods to foster discussion, experimentation and iterative adjustment of approaches. During participatory workshops, project team members learned that children were not benefitting much from increased food production because of women’s heavy workloads and because often men decided to sell the legumes rather than eat them, and used the money for alcohol [15, 31]. The project team, upon recommendation from farmer research team members (FRT) embarked on community-based participatory nutritional education intervention called “recipe days” to promote healthy feeding of complementary foods for under-five children, share skills on how to prepare diverse recipes, and to act as a platform to encourage more equitable household gender roles [56]. A transformative education approach was used with community members who took part in the recipe days. The recipe days were organized and facilitated by a Farmer Research Team and community promoters trained by SFHC^1^. In many communities, village heads or chiefs were also actively involved in organizing the events. Men and women came together to prepare and share recipes from the diverse range of local crops cultivated by farmers, as a way to not only teach nutritional skills, but encourage men to be more involved in child care and cooking [56, 57]. At each recipe day, emphasis was put on the important roles of men in childcare practices. Over time, the recipe days explicitly incorporated discussion topics on child care as well as household division of labour and decision-making, using a dialogue approach (as opposed to a ‘lesson’) in a lively session, often including some music and dance [56]. This transformational approach attempts to open up a discussion about emergent gender roles [58].

This study was carried out in nineteen villages around Edundu trading centre, 20 km away from Ekwendeni (Fig. 1). With the exception of a local primary school, the Edundu area lacks all forms of basic infrastructure, including health care services. The residents access primary health care services from Ekwendeni Hospital. The SFHC Farmer Research Team facilitated our entry into this study area. Nearly 300 households in the study area were already SFHC project participants but had not yet taken part in recipe days at the time of the study. The participants were self-selected into the project, but the village areas were purposively selected based on level of food insecurity and interest in project participation. We used purposive sampling [59] to select the Edundu area because it had not yet had any participatory nutrition education at the time of the study and was a relatively new project area of 2-3 years depending on the village (other areas had been involved for up to 12 years). In addition, it had high rates of food insecurity and gender inequality, according to key informant interviews.

## Methods

**Fig. 2:**
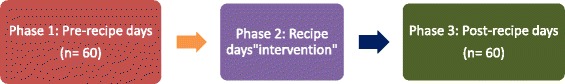
Research Design

Data were collected on the research participants’ reported experiences and perceptions on childcare and domestic work *before* and *after* the recipe day interventions took place (see Fig. 2). In each village, we obtained permission from chiefs before starting the research. We conducted four months of fieldwork between May and August 2012, and conducted 127 in-depth interviews with 67 people; 120 of the interviews were with 30 married couples with a child or children under age 5. Separate interviews were held with husbands and wives, on two separate occasions before and after the recipe days. The remaining 7 interviews were held with key informants, including village heads and elders. All the interview participants were active members of SFHC. There were two components to the interviews: a pile sort activity and semi-structured questions. Pile sorting is a structured data collection strategy used to understand how a particular community thinks about a particular domain [60–62]. Before commencing each interview, participants gave us a verbal consent because of high levels of illiteracy and suspicion of having to sign a document. When we were pre-testing the in-depth interview guide, participants opted for verbal consent to a written consent. All the verbal consents were communicated in the local language, chitumbuka. Respondents were asked to sort a pile of cards, each with a different household activity, into 3 categories: tasks that primarily husbands did, tasks primarily the wife did, and tasks that both did. The list of activities was generated with key informants prior to carrying out the interviews. The researchers then carried out an interview on decision-making and reasons for the division of labour. The aim was to produce consistent data which could be comparable within and across the households, and to identify patterns, common themes and perceptions for such division of domestic work. The pile sorting exercise was repeated with the same 30 couples after the recipe days had occurred.

We used an interview guide to initiate the discussions. In addition to demographic data, the interview guide contained questions on gender division of domestic work, perceptions of child health, parental nutrition knowledge, and factors influencing child care, dietary diversity and food security. The interview guide was pre-tested with 10 participants before the actual data collection. In order to moderate cross-gender sensitivities, one of the male authors conducted all interviews with men. We hired a trained female research assistant to conduct interviews with women. All couples were interviewed concurrently, but in locations not within earshot. Interview duration averaged 67 min, with a range from 52 min to 83 min. All interviews were conducted in Chitumbuka, a local language, and were tape recorded with permission from respondents. We received research ethics approval from the Non-Medical Research Ethics Board at Western University, Canada (Protocol Number: 18970S). We did not seek ethical approval specifically for this study in Malawi because it was a component of an ongoing large research project that got approval from National ethics committee and the Ekwendeni hospital in Malawi. We clearly indicated in our ethics application at Western University that the study was part of a large study that got approval from Malawian ethics committee. The in-depth interviews were complemented by participant observation in homes and during the recipe days. These methods allowed us to develop a nuanced understanding of communities’ everyday experiences with the participatory nutrition education [59]. Although our strategy was not aiming to be statistically representative, we drew upon a diversity of perspectives from a sample with enough variability based on age, gender, number of infants per married couple, and number of years of involvement with SFHC activities.

The audiotapes were first transcribed verbatim into Chitumbuka, and then later translated into English. To ensure continued immersion in the qualitative data [59, 63] the transcripts were analyzed using hand-coding. First, we read and reread the raw data line-by-line and then inductively derived codes relevant to our research questions [64]. Next, we organized and linked emergent codes into three broader themes. In order to assess the prominence of each theme, we also analyzed theme frequencies and the number of participants who articulated a particular theme [63, 65].

We used a number of strategies to ensure the rigour and validity of our qualitative findings [65]. To achieve reliable representation of the data, we interviewed a wide range of participants from each study village. We also used member-checking by sharing preliminary results at a feedback workshop held on August 16, 2012 at Ekwendeni Hospital. Although we invited and arranged transportation for all the original 67 in-depth interviews participants, only 41 attended the feedback workshop. The remaining 26 participants could not attend for several reasons, including farm work, child care, and illness. At the feedback workshop, we asked participants to comment on the accuracy of key themes derived from the transcripts. Participants clarified several concepts and this feedback was incorporated into the analysis and the final results. We have also ensured face validity by conveying respondents’ perceptions in their own words.

## Results

At the time of interview, participants ranged in age from 21 to 70 years. A total of 32 women and 35 men were included in the study. Each of the respondents had been involved in the “SFHC project” for an average of three years. Table 1 provides data on age groupings, marital status and educational background of the study participants.

**Table 1:** Socio-demographic characteristics of study participants (*n* = 67)

Variable	N (%)
Number of women interviewed	32 (48)
Number of men interviewed	35 (52)
Age	
*18–30*	25 (37)
*31–60*	30 (45)
*60+*	12 (18)
Marital status	
*Married*	64 (95)
*Single*	3 (5)
Education	
*No education*	26 (39)
*Some primary education*	19 (28)
*Completed primary education and beyond*	22 (33)
Number of children under age 5 in household	
*1 child*	33 (49)
*2 children*	26 (39)
*3 children*	8 (12)
Number of years participant has been involved in the recipe days and nutrition education	
*>5 years*	31 (46)
*1–5 years*	19 (28)
*<1 year*	17 (26)

Data source: Fieldwork, May to August, 2012

The study results are presented in two interdependent formats. The first is a theme-count table (Table 2), which shows the relative prominence of each emerging theme, as well as the number of participants who articulated a particular theme. The second is the use of exemplary quotations to show how participants attached meaning to each emerging theme. These quotations have been selected using the following criteria: (1) the ability to represent divergent perspectives; (2) typical views expressed by many respondents; and (3) the depth or clarity with which the ideas were conveyed. To protect confidentiality, our respondents are identified only by pseudonyms.

**Table 2:** Theme-count table

Basic themes identified	Organizing themes	Frequency in transcripts	Participants who mentioned theme (*n* = 67)
1. Appropriate gender roles for women and men 2. Tamed husbands 3. Love potion 4. Community disapproval of certain gender roles	• Social constructions of appropriate gender roles in child care	126 times	57 (85%)
1. Women’s enhanced control over resources 2. Improved understanding of gender relations	• Improved intra-household gender relations	103 times	53 (79%)
1. Increased legume consumption and dietary diversity 2. Improved knowledge about nutrition 3. The frequency of feeding different food groups	• Improved child care and feeding practices	134 times	61 (91%)
1. Co-learning involving all partners 2. A sense of local ownership of project 3. Intergenerational transfer of knowledge on local foods	• Community involvement and ownership of nutritional interventions	97 times	49 (73%)

Note: Basic themes are listed in a descending order of frequency of occurrence in the transcripts

Data Source: Table prepared following qualitative data analytical steps suggested by Baxter and Eyles [65], and Miles et al. [63]

### Social constructions of appropriate gender roles in childcare

Our results revealed a number of barriers that undermine the quality of child care in the research communities. Many of the factors mentioned were linked to gender constructions and norms on appropriate roles for women and men. Hegemonic masculinity in this context includes a notion that men are not supposed to take care of children. According to men who were interviewed, it was considered awkward for a man to be involved in child care, as it threatened their masculinity and brought feelings of shame and discomfort as a result. Hence, men mostly do not prioritize or feel the need to engage in child care, thus leading to women being solely responsible. In explaining why he doesn’t feel obliged to participate in child care, one male participant said:

The fact that I’m not supposed to take care of the children makes it sensible not to learn childcare skills. It can be awkward for me to learn a thing that I know will end up bringing misery, as community members will be laughing and teasing me. I don’t want to stoop so low in this village in a pretext of loving my wife [Ndiuzaani, Male, 26 years].

Ndiuzaani is expressing complicity with the hegemonic norm, in that he might be teased by others if he helps with child care. Attending under-five clinics was reported as one of the activities of childcare that men typically do not like to do, again related to hegemonic masculinity norms of maintaining respect and keeping separate from domestic spaces and subjects. The study participants gave several reasons why men do not like the idea of attending these clinics, particularly the discussion topics at the under-five clinics, including domestic work, child spacing and family planning. As one female participant explained:

I know the type of food to feed my children because I learn this at under-5 clinics. Men don’t attend these lessons because domestic work is mostly discussed over there, and men feel that these discussions do not concern them [Judith, Female, 28 years].

Another major issue, related to strong local taboos about being in the presence of your parents-in-law of the opposite sex, was that men felt it to be highly awkward to sit in under-five clinics with women, including mothers-in-law, and discuss issues related to child spacing and family planning. They also expressed concern that attending the clinics would result in them having decreased respect from other men, in keeping with the hegemonic masculinity of being separate from and above the domestic concerns of women and children. According to the men who were interviewed, attending an under-five clinic does not resonate well with hegemonic masculinity norms of what it means to be a real man. One male participant forcefully summarized these hegemonic concerns and the ways in which respect for him as a man was diminished in these female spaces:

I can’t go there (at the under-five clinics) because they treat participants as Standard one children. I can’t sit down with all these beards in my chin singing those silly songs. It is only an imbecilic man who can mix with women and freely start contributing to topics of child spacing amidst his mother-in-law and other elder women. Topics of sex are embarrassing especially when they are people that deserve respect from you [Yohane, Male, 37 years].

Hegemonic masculinities emerged as critical in discouraging men from being involved in child care. While many men and women expressed interest in helping with childcare practices, they indicated that *other* community members typically frowned upon women who allow their husbands to do so. The interview narratives suggested that women are also ridiculed if their husbands spend more time at home, or get involved in child feeding. Reportedly, community members would sometimes say a woman has ‘tamed the husband,’ or giving a man a ‘love potions,’ or, reminiscent of colonial masculinity narratives, put the husband ‘under petticoat governance.’ A male participant acknowledged the many difficulties faced by couples, especially when a man attempts to assist with childcare:

There are different reactions from people. Some just appreciate that the partners love each other. But *most* people mock the wife for using the herbs to tame her husband. Once the husband starts to spend more time at home, actively partakes in household chores, and consults his wife in decision making, many people believe that a wife has fed her husband, ‘khuzumure’ (love charm) [Faluzi, Male, 35 years].

Love potions have long been used by Malawian women to prevent infidelity, and keep men from migrating long distances, but the use of such potions are fraught with domestic tensions, linked to AIDS, unequal control over financial resources, women’s gendered responsibilities to produce children, and struggles with in-laws [66, 67]. There are many stories of the use of love potions leading to disastrous consequences for families, such as men going insane or being unable to work [67]. Thus both men and women who might consider emergent alternative masculinities are discouraged through community pressure, gossip and accusations that all reinforce hegemonic masculinities. The perception that husbands who do domestic work are not real men also came out explicitly from a woman who was doing a small scale business:

When I was in a business of selling beans at the trading centre, my husband was helping me in domestic work. Unfortunately, his friends kept on teasing him that he is ‘under petticoat government’, which means that I am the one who is heading the family. He confessed to me that he was eager to assist in domestic work but in order to save his face, he opted to quit [Pemphero, Female, 27].

Here again, while Pemphero’s husband expressed a willingness to try new roles, the hegemonic masculinity ideas imposed through persistent teasing of other men, led him to comply with these norms. While study participants acknowledged the critical roles that men could play in childcare practices, there was a continued sense that these community-level perceptions undermine men’s ability to be involved in such activities.

### Improved intra-household gender relations

All the 67 respondents reported that with the introduction of recipe days, there have been improvements in gender roles and responsibilities for childcare. The men described having learnt the crucial importance of childcare through discussions at the recipe days. They reported feeling an increased sense of responsibility for childcare and nutrition. It was also reported that there has been a greater reduction in community-level perceptions that frown upon women who allow their husbands to participate in childcare. Women respondents reported feeling happy to be able to call upon their husbands for help with respect to childcare. One woman expressed her joy by saying:

I’m now able to ask my husband to look after the child at home while I am doing some work. Previously, I couldn’t even try to request that because I was afraid he could complain to our marriage counsellors who could fault me. But now, he knows that his case can’t be taken seriously because the counsellors also learnt the importance of gender during the recipe day [Navess, Female, 33 years].

Men expressed satisfaction that they could more freely assist in childcare without any disparaging looks from community members, evidence that new ‘emergent masculinities’ were developing. They expressed a deep sense of pride to be able to build a caring relationship with their infant babies, and watch them grow healthy. A middle-aged man used a proverb to summarize his experience with the recipe days:

I can summarize what I learnt from the recipe day sessions using the following idiom; “A child can die of thirst while standing in a pool of water”. What I meaning is that a child can still suffer from undernutrition even if there is a lot of food in the household. To avoid our children suffering from undernutrition, I’m assisting my wife to feed the child and the baby is eating a wide variety of foods in a single day [Mavuto, male, 37 years].

Since the recipe days started, men said they have afforded a greater opportunity to understand how women’s multiple roles affect child nutrition. This sentiment was vividly conveyed by a male participant:

Recipe day education was clear and appealing that if I and my wife are not going to assist each other in caring for the children, then there are still high chances that the child can suffer from malnutrition. I’m now able to link negative child health outcome with women’s multiple roles; a thing that I was not aware of at first [Likambale, Male, 32 years].

Both women and men said they now have a comprehensive understanding of child health and nutrition, including the importance of breastfeeding and complementary feeding. Besides the greater involvements of men in child care, participants also mentioned increased decision making and a shift in control of household resources towards women. Women’s lack of access to food resources has often been a major problem affecting food preparation, meal content and child feeding in rural northern Malawi [15]. Following participation in the recipe days, participants reported that this problem is gradually waning as one woman noted:

Recipe days education has acted like love potion to my husband, he is now allowing me to take whatever household resources are required in order to prepare food for the family. He too apologised to me that he was not aware that he was contributing to ill health of our family when he was monopolizing the resources [Linda, Female, 38 years].

For women, it was highly fulfilling that through the recipe days, there is a growing understanding of the importance of fathers’ time in infant and child care. It was equally fulfilling that men were beginning to appreciate the importance of equal decision-making, especially on issues that affect child growth and development. For women, it was fulfilling to them that they had increased control over resources to both secure sufficient quality food and give it to children according to need. These different dimensions of changed attitudes to gender roles suggest the possibility of new ‘emergent masculinities’ described by Inhorn and Wentzell in Mexico and the Middle East as ‘ongoing, context-specific, embodied changes within men’s enactment in masculinity’ ([68]:823).

The pile sort activities that couples carried out suggest that a small proportion of households were carrying out changes in the division of labour (Tables 3 and 4). Results from Table 3 shows that 13% of respondents (four couples) indicated that husbands were now involved in drawing water from the well.

**Table 3:** Pile sorting of child care activities (*n* = 60 respondents)

	Pre-recipe days			Post-recipe days		
Childcare activities	Mainly wives (%)	Mainly husbands (%)	Done almost equally (%)	Mainly wives (%)	Mainly husbands (%)	Done almost equally(%)
Bathing child	60 (100)	-	-	58 (97)	-	2 (3)^a^
Changing diaper	60 (100)	-	-	60 (100)	-	-
Cooking for child	60 (100)	-	-	54 (90)	-	6 (10)^a^
Feeding child	60 (100)	-	-	52 (87)	-	8 (13)^a^
Doing laundry	60 (100)	-	-	58 (97)	-	2 (3)^a^
Playing with child	60 (100)	-	-	7 (12)	1 (1.7)	52 (87)^a^
Taking child to under-five clinic	60 (100)	-	-	57 (95)	-	3 (5)^a^
Going to hospital with the child	15 (25)	-	45(75)	10 (17)	2(3)	48 (80)^a^
Nursing sick child	58 (97)	-	2 (3)	55 (92)	-	5 (8)^a^

^a^indicates reported change in practice

**Table 4:** Pile sorting of household tasks (*n* = 60 respondents)

	Pre-recipe days			Post recipe days		
Household Task	Mainly wives (%)	Mainly husbands (%)	Done almost equally (%)	Mainly wives (%)	Mainly husbands (%)	Done almost equally (%)
Laundry	60 (100)	-	-	56 (93)	-	4 (7)^a^
Herding livestock	-	60 (100)	-	-	60 (100)	-
Constructing pit latrine	-	60 (100)	-	-	60 (100)	-
Shopping	11 (18)	12 (20)	37 (62)	-	-	60 (100)^a^
Ironing	-	13 (21)	47 (70)	-	8 (13)	52 (87)
Sweeping	48 (80)	-	12(20)	27(45)	2(3)	31 (52)
Cooking	60 (100)	-	-	54 (90)	-	**6 (10)** ^a^
Farming	-	12 (20)	48 (80)	-	4 (7)	46 (93)
Pounding maize	60 (100)	-	-	60 (100)	-	-
Caring for livestock	-	19 (31)	41 (68)	-	-	60 (100)
Fetching firewood	60 (100)	-	-	58 (97)	-	**2 (3)** ^a^
Earning money	-	36 (60)	24 (40)	-	-	60 ()
Going to maize mill	56 (93)	-	4 (7)	48 (80)	2(3)	**10 (17)** ^a^
Drawing water	60 (100)	-	-	52 (87)	-	**8 (13)** ^a^
Washing Dishes	60 (100)	-	-		58(97)	-

^a^indicates reported change in practice

**Fig. 3:**
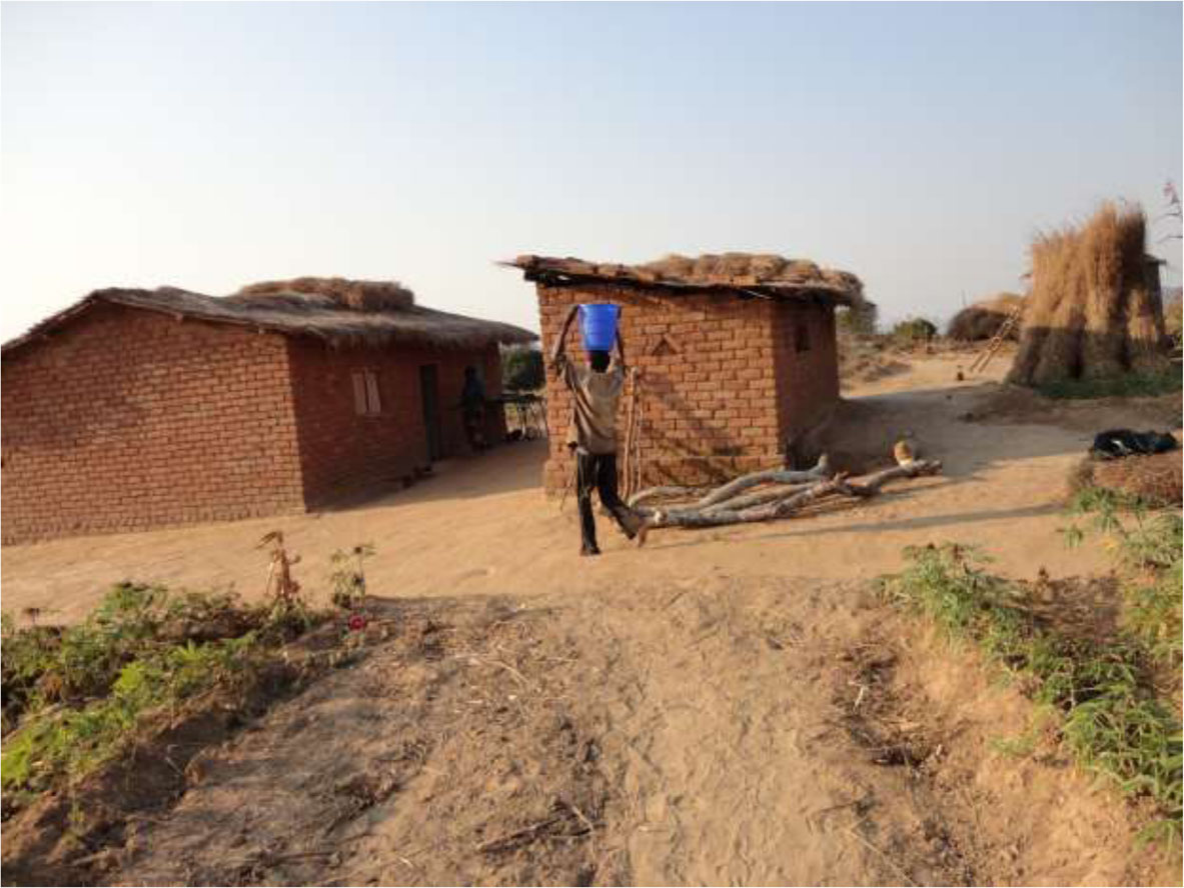
A husband carrying a water bucket

While reported changes in practice alone are not adequate evidence, unscheduled visits to the villages in the months that followed by the authors confirmed that at least some men had begun to cook, help with child care and carry water (Fig. 3), showing a significant change from the past. When one man who was carrying water approached, he said:

You do not need to ask people if we are implementing what we learnt at recipe day education. You have seen with your naked eyes [Timothy, male, 30 years].

During subsequent visits to other villages, men and boys were seen carrying water using wheelbarrows or on their heads.

### Community involvement and ownership of nutrition interventions

The study participants highlighted the potential for success when interventions are carefully designed to meet local needs. They greatly appreciated the participatory nature of the project, as well as the explicit and inclusive focus on both men and women. According to comments from the interviews, farmers mentioned key characteristics that have contributed to the success of the work, and that also make this approach unique. These include an all-encompassing focus on sustainable agriculture, combined with social aspects such as gender and intensive nutrition education. As one male participant emphasized:

That was my first time to be involved in education that discusses about gender, child nutrition and food preparation. If all the education were designed in such a way, I hope all people could by now understand the meaning and importance of ‘jenda’ in the households. The problem with other approaches is that only women are called to learn about ‘jenda’ forgetting that it is an issue of men and women [Gono, Male, 35 years].

This approach is a unique aspect that farmers reportedly said was lacking in other agricultural intervention programs. Due to the participatory and community-based co-learning approach, study participants said they feel a deep sense of owning the project. Farmers commented positively on how leadership roles are assigned in the project, the inclusion of village heads, and a greater respect for community views. The role of male village leaders was mentioned by some participants as crucial in encouraging reshaped masculinities, one that allowed men to discuss and be concerned about formerly female-only spaces and subjects such as child care and nutrition. They contrasted this approach with other agricultural and nutrition intervention programs in Malawi, which did not take local norms into account, or which overemphasized either nutrition or agriculture. This contrast emerged strongly in the comments of one respondent:

The fact that we were involved at the beginning of the program, we felt like we owned it. In this regard, you find out that we keep on brainstorming ways of improving our health status. It’s different from other autocratic projects that they just tell us what to do [Nabanda, Female, 25 years].

Farmers also appreciated the focus not only on mainstreaming gender into agricultural and nutrition interventions, but also fostering intergenerational learning. Among respondents who were above age 50, they often mentioned how the recipe days are fostering the sharing of traditional food knowledge between the young and elderly. As one village elder explained:

I attended two of the recipe day sessions; I appreciated how the participants incorporated us (old people). They were giving us chance to demonstrate how to make our forefathers’ foods such as Chiponde (peanut butter) and Nkhowe (shepherd pie). We used to eat these foods on our way on foot to South Africa. We were so healthy and energetic by then, but alas! Current generation don’t have idea of such foods [Elder, Male, 70 years].

The elderly talked extensively about how the recipe days have brought to the fore the health benefits of traditional foods. They expressed a great sense of willingness to continue participating in community nutrition education, so that local food values are retained and transmitted from one generation to another. These continuities in local cultural knowledge provided new hybrid spaces that linked new emergent masculinities and historical food ways of male migrants.

## Discussion

The quality of care giving that children receive in the first 36 months of their lives is crucial, because food habits and health status at this formative stage could have long-term implications for proper growth and development. While improved agricultural practices have a great potential to increase incomes, the quality of household diets, and the consumption of different food groups, research has shown that these are not enough to address undernutrition [1, 69]. Instead, efforts should be made to purposefully transform the socio-cultural context of caregiving [1, 3, 70]. These efforts may include the use of nutrition and health education, as well as transformative learning programs. In this study, the project promoted some of these transformative learning programs together with sustainable agriculture in northern Malawi over the past ten years. This evaluative case study of the SFHC project highlights the importance of making agricultural programs nutrition sensitive.

For many households, the social context of child care is as important as the quality and quantity of the food that is available for consumption. Even when food resources are available, access to and control over these resources and gendered power relations could affect food preparation, nutritional content and the overall quality of child feeding [71]. Moreover, child care capacity could be compromised if left on the shoulders of a few household members, especially women who are already overburdened with both domestic and agricultural work [1, 71]. Findings from our study show that participatory agriculture and nutrition education that explicitly addresses hegemonic masculinities that affect child nutrition, such as ideas about female-only spaces and women’s roles in child care, can begin to change these gender norms. A key aspect of the emergent masculinities is involving male leaders and integrating agriculture and food security concerns with nutrition. In doing so, men’s concern with respect is both adhered to and altered, which speaks to the hybrid, fractured and contingent masculinity at work in Malawi [20]. With the use of community-based participatory education on nutrition, health and gender, the study participants have seen improvements in many social factors that affect child care. For example, men in the study villages are increasingly being involved in previously perceived “feminine” domestic work, including complementary feeding and cooking. These findings are in line with other studies that have also explored the impacts of agricultural interventions on nutrition [3 70, 72]. For example, Ruel and Alderman [70] have reviewed the literature in this field and concluded that agricultural programs have an impact on nutrition when behavior change and empowerment activities are included.

## Conclusion

This study sheds light on different methods by which new gender norms can be fostered, through community-based educational activities that include both women and men. The active involvement of maternal and paternal grandparents is crucial because they are important decision-makers when it comes to child care [31]. For local acceptance and long-term sustainability in reshaping masculinities, such programs should involve community stakeholder including village chiefs and elders. In the particular case of Malawi, culture and community-level perceptions shape some of the hegemonic masculinities related to responsibilities for child care and domestic work. It is precisely for this reason that communities rather than individual households should be involved in nutritional education programs. This research has also shown that participatory nutrition education has the potential of bringing together community members of diverse age groups to share not only recipes on local foods, but also to discuss the significance of household gender equality on child health.

While our research provides critical insights on how to make agriculture and nutrition interventions effectively address gender norms, certain study limitations should be acknowledged. As an in-depth qualitative research, our results should be interpreted in context. Given our sample size (*n* = 67 respondents) and sampling strategy, our findings cannot be considered representative of the experiences of all households or villages in northern Malawi. Indeed, our main aim was not to study a larger sample and generalize our findings, but rather, to provide an in-depth account of the experiences of a smaller sample participating in SFHC interventions. In this respect, we endeavoured to obtain a diverse range of experiences from a sample that varied based on age, gender, educational level, number of children under age 5, and number of years of participation in SFHC’s interventions (see Table 1). All our interview themes also reached theoretical saturation, meaning that interview numbers were sufficient enough to draw conclusions among the sample population [63]. This limitation notwithstanding, the present qualitative study sets the groundwork for future quantitative research to test the representativeness of the findings reported here. A second major limitation is the possibility of response bias, since the authors might be associated with SFHC and thus respondents might tell us what was heard at the sessions. We tried to limit this bias by clearly explaining the purpose of the study to participants, and by using different probes and types of data to check for validity. The observations made during unscheduled visits to the participants after the recipe days provide some evidence of the validity of the results. Further research, using a broader range of methods and a larger sample size, are needed to generalize these findings.

Despite these limitations, this study provides some strategies to move beyond a ‘crisis in masculinity’ and towards emergent masculinities that, however tentatively, embrace a new man, one who cooks, helps his wife and cares for young children. This new masculinity is fraught with contradictions, tensions and struggle, but even in the highly vulnerable and challenging context of rural Malawi, is possible. Key to this approach, in our view, is transformative participatory methods that integrate gender issues and new emergent masculinities into broader agriculture-nutrition approaches, in culturally respectful and sustainable ways.

## Abbreviations

AIDS: Acquired immunodeficiency syndrome
FRT: Farmer research team members
MAFFA: Malawi Farmer-to-Farmer Agroecology project
SFHC: Soils, Food and Healthy Communities project

## Footnotes

1 SFHC is now a farmer-led non-profit trust organization, and collaborates with the University of Malawi, Chancellor College and several other universities to implement the Malawi Farmer-to-Farmer Agroecology project, which involves over 6000 farming households in central and northern Malawi. This paper reports on research conducted prior to the implementation of MAFFA.
